# PH-dependent cell–cell interactions in the green alga *Chara*

**DOI:** 10.1007/s00709-019-01392-0

**Published:** 2019-07-31

**Authors:** Alexey Eremin, Alexander A. Bulychev, Christopher Kluge, Jeremy Harbinson, Ilse Foissner

**Affiliations:** 1 Institute of Physics, Otto von Guericke University of Magdeburg, 39016 Magdeburg, Germany; 2 Department of Biophysics, Faculty of Biology, Moscow State University, Moscow, 119991 Russia; 3 Department of Plant Sciences, University of Wageningen, 6708 PB Wageningen, The Netherlands; 4 Department of Biosciences, University of Salzburg, 5020 Salzburg, Austria

**Keywords:** Characean internodal cells, Charasomes, Kinetics of alkaline band formation, Mitochondria, Mutual interactions, pH banding pattern, Photosynthetic activity Y(II)

## Abstract

Characean internodal cells develop alternating patterns of acid and alkaline zones along their surface in order to facilitate uptake of carbon required for photosynthesis. In this study, we used a pH-indicating membrane dye, 4-heptadecylumbiliferone, to study the kinetics of alkaline band formation and decomposition. The differences in growth/decay kinetics suggested that growth occurred as an active, autocatalytic process, whereas decomposition was due to diffusion. We further investigated mutual interactions between internodal cells and found that their alignment parallel to each other induced matching of the pH banding patterns, which was mirrored by chloroplast activity. In non-aligned cells, the lowered photosynthetic activity was noted upon a rise of the external pH, suggesting that the matching of pH bands was due to a local elevation of membrane conductance by the high pH of the alkaline zones of neighboured cells. Finally, we show that the altered pH banding pattern caused the reorganization of the cortical cytoplasm. Complex plasma membrane elaborations (charasomes) were degraded via endocytosis, and mitochondria were moved away from the cortex when a previously acid region became alkaline and vice versa. Our data show that characean internodal cells react flexibly to environmental cues, including those originating from neighboured cells.

**Electronic supplementary material:**

The online version of this article (10.1007/s00709-019-01392-0) contains supplementary material, which is available to authorized users.

## Introduction

**Fig. 1:**
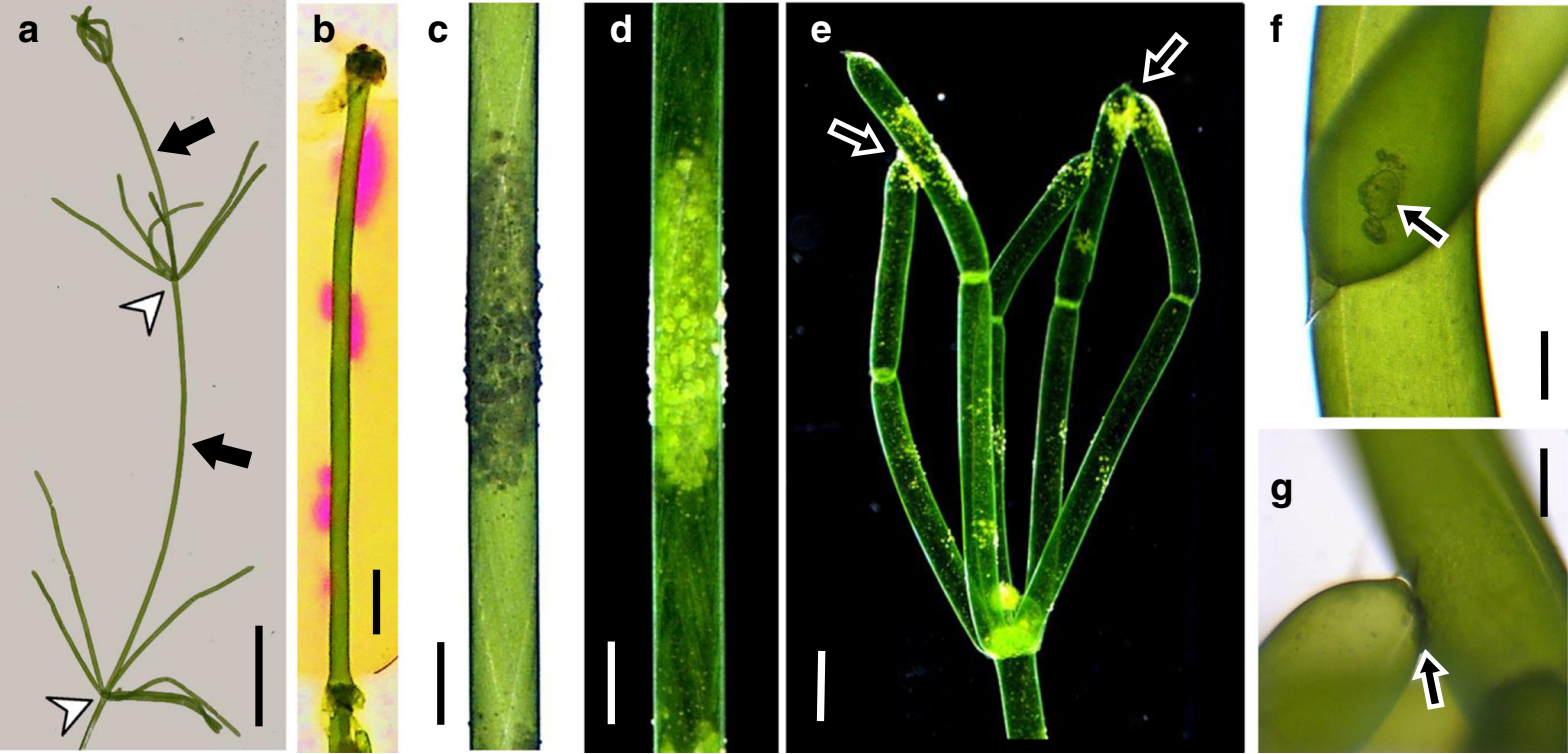
Thallus and internodal cells of *Chara australis*. **a** The characean thallus consists of internodal cells (arrows) separated by groups of smaller nodal cells (arrow heads). Branchlets (series of shorter internodal cells) extend from the nodes. **b** PH banding pattern of an internodal cell visualized by phenol red. Pink colour indicates alkaline pH. **c**, **d** Calcified cell region in bright field (**c**) and in dark field microscopy (**d**). **e** Tip of a thallus (dark field microscopy). Arrows indicate CaCO_3_ deposits agglutinating the tips of branchlet internodal cells. **f**, **g** CaCO_3_ deposits (arrows) between branchlet internodal cells from above (**f**) and from the side (**g**). Bars are 1 cm (**a**), 2 mm (**b**), 1 mm (**c**–**e**), 150 μm (**f**, **g**)

The multicellular characean green algae are a long established group of the plant kingdom and closely related to higher plants (Wickett et al. 2014; Nishiyama et al. 2018; and references therein). Their thallus consists of groups of small nodal cells and huge internodal cells, which may attain a length of up to several centimeters (Fig. 1a). In their aqueous environment, carbon, required for photosynthesis, is mainly present as HCO_3_^−^ (hydrogen carbonate) which is poorly membrane permeable. In order to overcome the diffusion limitation, characean internodal cells locally acidify their surface. The lower pH facilitates the conversion of HCO_3_^−^ to membrane-permeable CO_2_ either directly or via carbonic anhydrase, thereby increasing photosynthetic efficiency at the acid zones (Price and Whitecross 1983; Price and Badger 1985; Price et al. 1985; Plieth et al. 1994; Bulychev et al. 2001b; Ray et al. 2003). In order to maintain pH homeostasis of the cytoplasm, proton efflux is balanced by either influx of H^+^ or efflux of OH^−^. The resulting pattern of alternating acid and alkaline regions (Fig. 1b) is known as pH banding and has been the subject of numerous studies (reviewed by (Beilby and Bisson 2012; Beilby and Casanova 2014). The internodal cell thus serves as a convenient model for investigating locally separated ion fluxes which play important roles also in carbon uptake of leaves of higher water plants (Elzenga and Prins 1989), in nutrient uptake by roots (Raven 1991) and during growth of pollen tubes (Hepler et al. 2013) and root hairs (Monshausen et al. 2007). The recent publication of the *Chara braunii* genome (Nishiyama et al. 2018) and the application of proteomic methods (Pertl-Obermeyer et al. 2018) will boost further research in this valuable model organism.

Whereas acidification is known to be due to the higher abundance and/or activity of a plasma membrane H^+^ ATPase, the nature of the carrier(s) involved in alkalinization is still under debate (Beilby and Bisson 2012). The so-called high pH channels (Bisson and Walker 1980) are likely candidates, but their identity has not yet been proven (see discussions in Absolonova et al. 2018a; Pertl-Obermeyer et al. 2018).

The pH banding pattern has hitherto been visualized with the aid of conventional pH, indicating dyes like phenol red (Fig. 1b) or with fluorescent fluorescein isothiocyanate (FITC) coupled to dextran (Absolonova et al. 2018a). FITC can be used for ratio imaging in the range between pH 5.0 and 7.5. Since the pH at the alkaline regions rises up to 10, meaningful quantitative measurements are only possible with the aid of ion specific microelectrodes (e.g. Fisahn et al. 1989; Bulychev et al. 2001a). Both microelectrode and fluorescent dye measurements have shown that alkaline spots grow and coalesce to form the alkaline bands (Bulychev et al. 2003; Absolonova et al. 2018a). The disadvantage of these two methods is that the changes in the pH can only be measured in the medium outside the cells. During this study, we used a fluorescent dye, 4-heptadecylumbiliferone, which monitors the pH at the plasma membrane.

The consequence of external alkalinization is the deposition of CaCO_3_ (Raven et al. 1986) and other mineral species at the cell wall (McConnaughey and Falk 1991; Schöler et al. 2014). In dense stands, neighbouring cells often stick together via calcium carbonate and the common encrustations suggest that cells interact with each other via local differences in external pH when in close contact (Fig. 1e–g). The aim of the study was to clarify how this interaction is achieved and how metabolism and cell structure are affected. Specifically, we were interested in the photosynthetic patterns of aligned cells because of the correlation of the pH banding pattern with photosynthetic activity (Plieth et al. 1994; Bulychev et al. 2001a).

We also investigated the effect of pH band interaction on the organization of the cortical cytoplasm with focus on the distribution of charasomes and cortical mitochondria. Charasomes are convoluted (3D or “cubic”) plasma membrane areas with a size of up to several micrometres, and their main function appears to provide space not only for H^+^ ATPases (Schmoelzer et al. 2011 and references therein) but also for other transporters (Franceschi and Lucas 1982; Pertl-Obermeyer et al. 2018). Under steady-state conditions, charasome size and abundance are high at the acid regions and low at the alkaline bands in cells with a stable pH pattern (Absolonova et al. 2018b and references therein). Charasome distribution is similar to that of the cortical mitochondria, which, among other functions, detoxify harmful metabolites produced during photosynthesis and power the activity of plasma membrane H^+^ pumps (Schmoelzer et al. 2011).

We found that characean internodal cells interact mutually via local pH differences (gradients) and that this interaction involves rapid changes in the photosynthetic activity of chloroplasts. Long-term interaction leads to displacement of mitochondria and growth/or degradation of charasomes. These data impressively show how alterations in physiological parameters affect cytoarchitecture even in fully grown, mature internodal cells. During the course of this study, we wanted to get more insight into the formation of alkaline bands and to clarify how cells interact with each other via local differences in surface pH.

## Results

### PH banding pattern and calcification

Figure 1 shows the thallus of *Chara australis* (Fig. 1a) and the pH banding pattern of an internodal cell visualized by phenol red (Fig. 1b). Under appropriate conditions, CaCO_3_ and other precipitates (Schöler et al. 2014) are deposited at the alkaline regions of the cells (Fig. 1c–g). Adjoining cells often share these encrustations, thereby sticking together. Fig. 1e–g shows how tips of branchlet internodal cells agglutinate. Internodal cells of the main axis can also interact with each other along their lateral surfaces and produce common calcified shields (not shown).

### Spatio-temporal dynamics of band formation

In order to visualize the nucleation of the pH patches and the growth of the pH bands at the level of the plasma membrane, cells were labelled with the pH-sensitive dye 4-heptadecylumbiliferone and were incubated in the dark for 30 min. During the measurement period, the cell was exposed to overall white light of a tungsten lamp (10 mW cm^−2^) interrupted only by the fluorescence image acquisition with the exposure time < 300 ms.

**Fig. 2:**
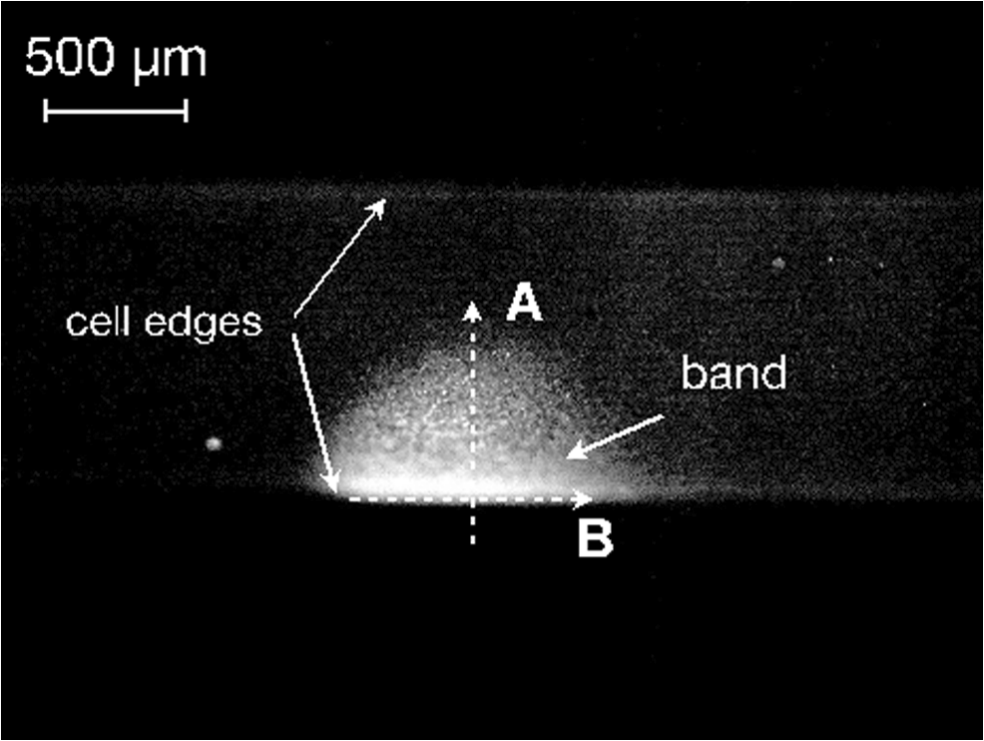
Fluorescence microscopy image of a cell treated with the pH-sensitive dye 4-heptadecylumbiliferone exposed to actinic illumination provided by a tungsten lamp for 12 min. The growing pH band has a higher intensity indicating a higher surface pH value than in the rest of the cell. The intensity profiles were analysed along the paths marked A and B

**Fig. 3:**
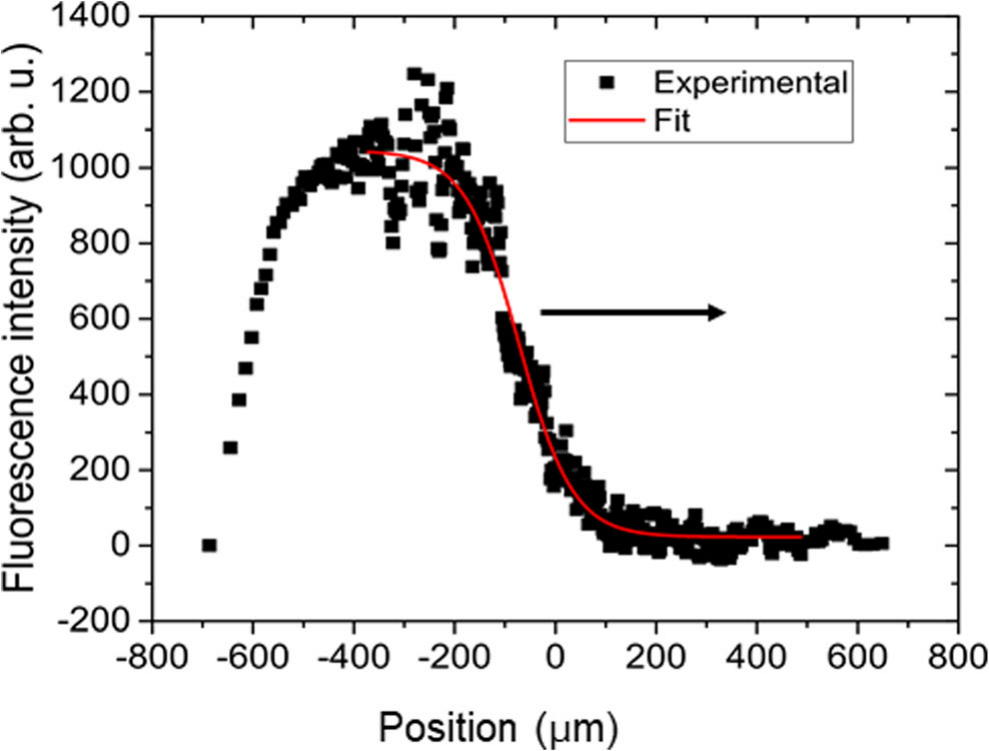
Intensity distribution across the profile A in Fig. 2. The intensity was corrected to account for the curvature of the cell. The red line is the fit with Eq. 1. The arrow shows the propagation direction

Figure 2 shows a pH patch, and its growth is illustrated in Fig. S1. To study the dynamics of the patch growth, we recorded the intensity profiles perpendicular and parallel to the boundary of the patch (profiles A and B, respectively) at time intervals of 20 s over 12 min. To account for the curvature of the cell, we normalized the measured intensity by the geometrical factor (corresponding to the intensity of a uniformly emitting cell). The normalized intensity was obtained as a function of a coordinate (*x*) of the projection of the cell to the object plane of the microscope. Spatial correction was introduced to map the projection to a coordinate system (*s*) bound to the cell surface. The spatial intensity distribution across the patch, corrected to account for the cell curvature, can be well described by a sigmoidal function (Fig. 3):

1 $$ I(x)=\frac{I_{\mathrm{max}}}{1+\exp \left(\frac{x-{x}_0}{w_0}\right)} $$

where *I*_max_ is the intensity maximum, *x*_0_ is the position of the patch front (inflexion point at the half-maximum) and *w*_0_ is defined as the front width. The position of the front as well as the maximal intensity are functions of time (for details see Supplementary Method). In case of the profile A, Eq. 1 is valid for the “unfolded” coordinate *x = s* along the cell surface (Fig. 3). The position *s* = 0 in Fig. 3 corresponds to the middle point in the projection of the cell on the observation plane.

**Fig. 4:**
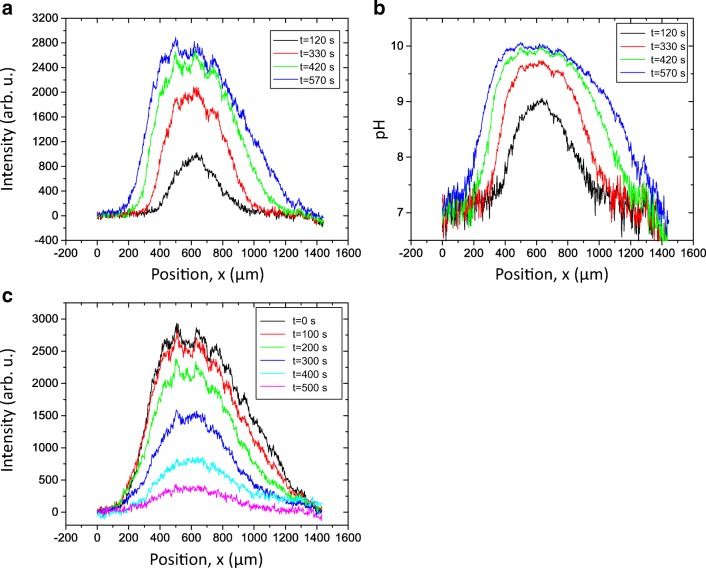
The intensity distribution profiles measured along the line B in Fig. 2 for the growth of the patch (**a**) and its decomposition (**c**). The pH distribution (**b**) can be estimated from the intensity distribution (**a**) using calibration with buffers

As the front propagated, the intensity was growing reaching a saturation value. To analyse the kinetics of the patch growth, it is more convenient to use the profile B (Fig. 2) where the intensity is significantly higher. In this case, the section of the patch is given by a bell-shaped curve (Fig. 4).

We can describe the development of a localized patch as a superposition of two fronts propagating in the opposite directions. Comparing the profiles (Fig. 4a, c) for the growth and decomposition, we can see that they exhibit different kinetics. The pH distribution can be estimated from the intensity distribution (A) using calibration with buffers (Fig. 4b).

**Fig. 5:**
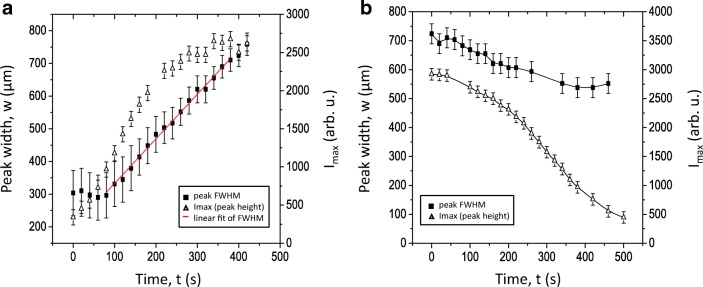
Growth and decomposition kinetics of a pH patch. The dependences of the full width at half maximum (FWHM) of an intensity profile in Fig. 4 (filled symbols) and the maximal intensity *I*_max_ (open symbols) for growth under actinic illumination (**a**) and decomposition in the dark (**b**)

The front velocity can be estimated from the time dependence of the width of the patch measured at half-maximum (FWHM) which is shown in Fig. 5. The growth dynamics exhibited three regimes. In the first regime, the patch area was localized, while the fluorescence intensity/pH increased. After the pH reached a certain value, the patch started growing transversally increasing in width. The pH front propagated with a nearly constant velocity (Fig. 5a). In the third regime, the growth slowed down and stopped. The velocity of the front in the linear regime was about 1.6 μm s^−1^ and remained constant for 7 min. Interestingly, the pH reached the stationary regime after about 4 min of the illumination. It happened before the systems left the linear regime.

Relaxation dynamics of the pH bands and patches are demonstrated in Fig. 5b (compare Fig. S1). In contrast to the growth dynamics, the relaxation occurred with relatively small displacement of the pH front. The width of the patch changed by approximately 20% over a period of time when the maximal intensity completely relaxed. This difference in growth/decay kinetics suggests that the growth occurs as an active, autocatalytic process, whereas decomposition is governed by the diffusive relaxation.

### Band interactions

**Fig. 6:**
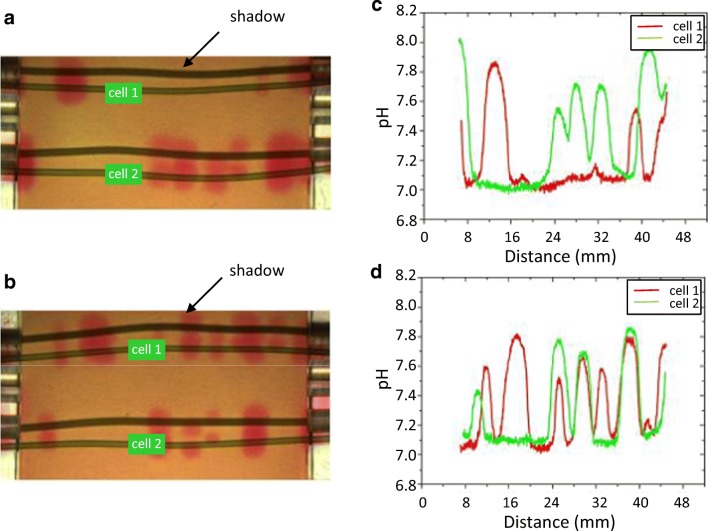
Interactions between pH bands of two neighbouring cells. The banding pattern was recorded in phenol-red containing solutions of AFW-NaHCO_3_ before (**a**) and after (**b**) incubation under actinic light for 30 min (cells were separated from each other after this incubation). The corresponding pH plots are shown in **c** and **d**. Note that in this figure and in Fig. 8, shadows are seen in addition to the original images of the cells

The interactions between the bands of the neighbouring cells were investigated in solutions of artificial fresh water (AFW)-NaHCO_3_ and the phenol red pH indicator. Pairs of cells exhibiting different band patterns were placed in close contact and incubated under actinic light for 30 min. At the end of the incubation period, the cells were separated and the pH banding pattern was recorded. Figure 6 shows the pH patterns and the corresponding pH plot of a cell pair before (Fig. 6a, c) and after the incubation (Fig. 6b, d). The two cells showed very different patterns before the incubation (Fig. 6a, c). After the incubation, additional bands appeared in the upper cell 1 and some bands become suppressed in the lower cell 2 (Fig. 6b, d).

However, the two patterns were not identical. Generally, in these experiments, we observed pattern matching accompanied by (1) band induction, (2) band suppression and (3) band displacement. The pattern matching occurred in both affected cells. None of the cells dominated the matching.

**Fig. 7:**
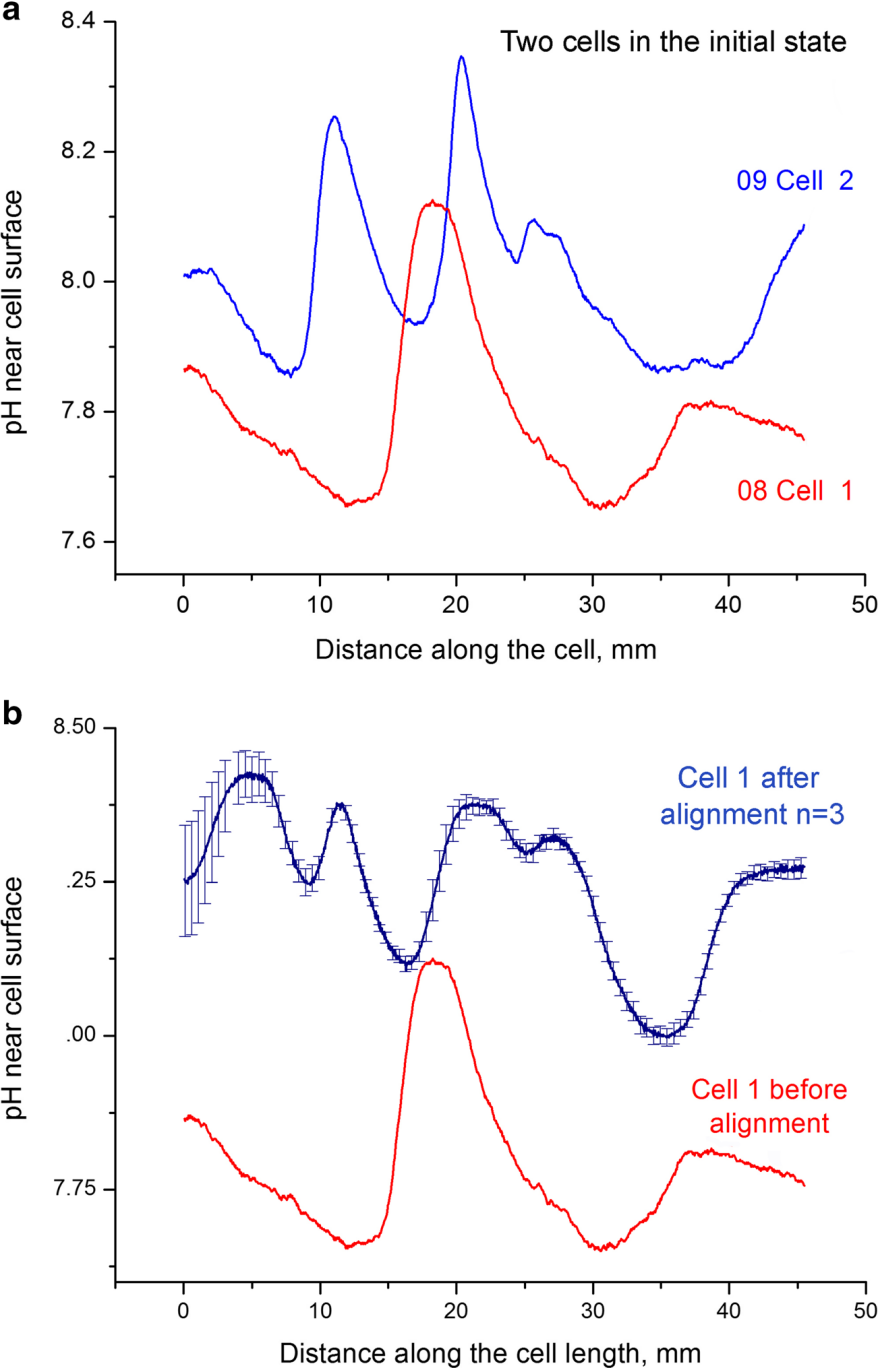
Longitudinal pH profiles of two *Chara* internodes. Cells were positioned parallel **a** at a 1 cm distance and then **b** were allowed to stay for 1 h under the same orientations and positions except that the distance between the internodes was narrowed to 0.5–1 mm. The irradiance was 40 μmol quanta m^−2^ s^−1^. The pH profiles were measured with antimony pH microelectrodes moved at a constant velocity relative to the cell

Intercellular interactions mediated by proton concentrations in the unstirred layers surrounding isolated internodes of *Chara* were also demonstrated with a scanning pH microelectrode. The technique was similar to that described in Bulychev et al. (2001a). The two internodes—cell 1 and cell 2—were initially placed parallel to each other at an intercellular distance of ~ 1 cm. The longitudinal pH profiles were measured at the velocity of pH microelectrode movement of 100 μm s^−1^. The initial profiles of pH along the widely separated cells are shown in Fig. 7a. They had stable shapes but differed in the number and positions of alkaline bands. Next, the distance between the cell was reduced without axial displacement of cells, and the pH profiles along cell 1 were measured repeatedly after allowing the cells to stay at a close proximity over 1 h. After the intercellular distance became narrow, the pH profile could be measured only for one cell on the side that remained accessible for the microelectrode inclined at a low angle to the horizontal plane. The longitudinal pH profile of cell 1 was modified substantially after this treatment (Fig. 7b). This result is similar to the observations obtained with a pH-indicating dye, phenol red (Fig. 6). It is consistent with the supposed chemical (pH-mediated) interaction of closely positioned cells.

### Effect of external pH on band induction

PH pattern matching could be due to changes in external pH. To investigate the role of the external pH, we made two kinds of experiments: (1) buffering a part of the cell in a high-pH medium and observing the band formation and (2) exposing cells to a pH pattern formed by a different cell. For these experiments, phenol red was used as pH indicator.

**Fig. 8:**
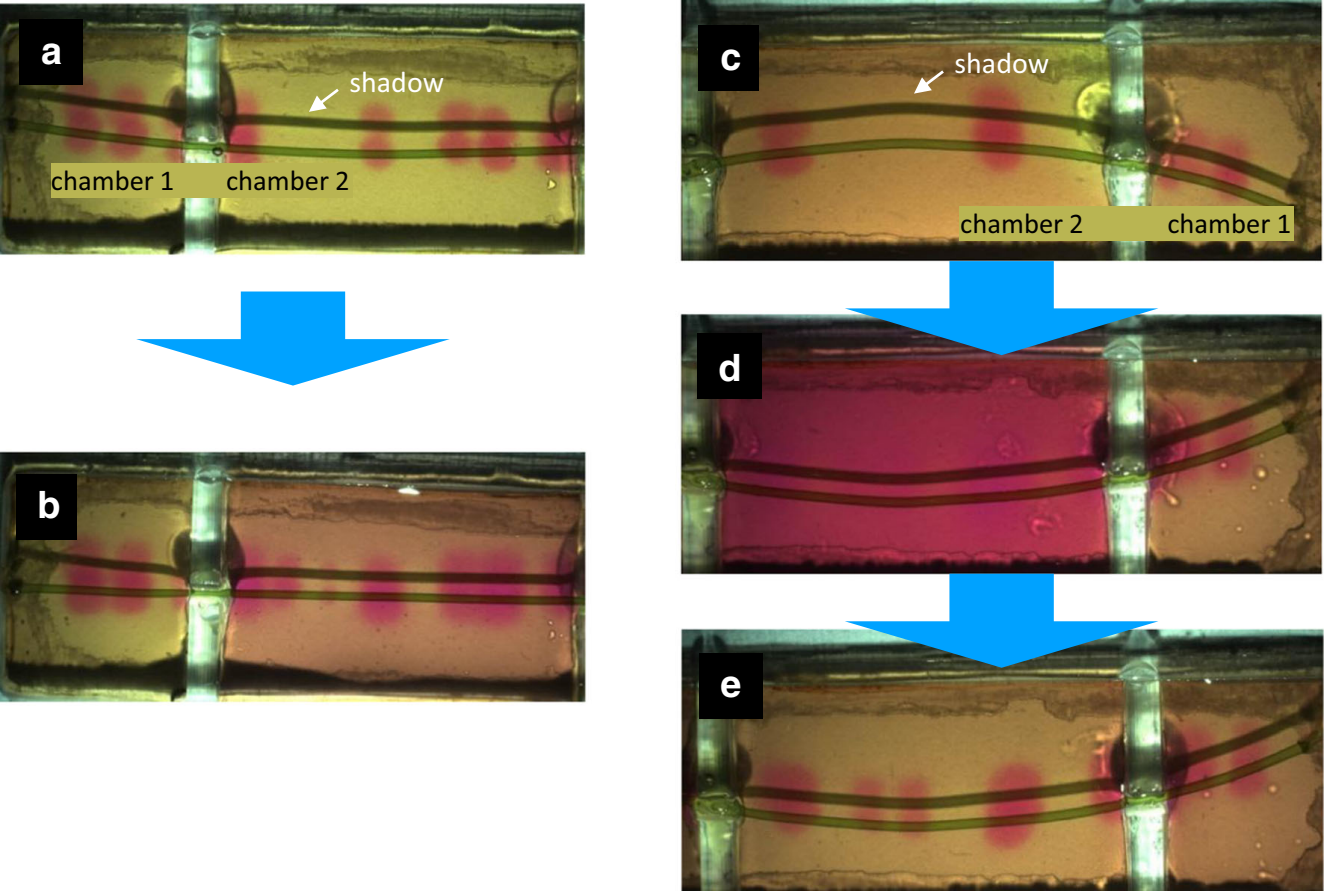
Experiment with a two-chamber cell holder. **a** The initial state with pH 6.76 in both chambers. **b** pH pattern after 30 min exposure with the pH 6.75 in chamber 1 and pH 7.21 in chamber 2. Yellow bands in chamber 2 correspond to the acidic regions. In the second experiment, the initial configuration is given in **c**. After two exposures to pH 8.12 in chamber 2 (**d**) and the following reduction of pH to 7.0, the number of pH bands increased (**e**)

The first experiment was performed in a two-chamber cell holder. In the first chamber, the pH far from the cell was kept at 6.76, and in the second chamber, it was increased to 7.21. As shown in Fig. 8a, b, there was no significant change in the pH pattern in the chamber 1 after 30 min exposure. At the same, the new bands appeared in the chamber 2 with a higher pH. The formation of additional bands was particularly well seen when the experiment was repeated several times. The initial state in Fig. 8c is characterized by a pair of pH bands in the chamber 2. After the first increase of the pH, a third band appeared (not shown). After the second exposure to pH 8.12 for 30 min (Fig. 8d) and reducing the pH back to 7.0, we found a pattern consisting of five bands, which was stable for several hours (Fig. 8e). Also, in this experiment, no significant change in the chamber 1 was observed.

This shows that an increase of the external pH leads to an induction of additional bands. In total, 12 cells were investigated with similar results.

### Changes in photosynthetic activity following band interaction and following increase in external pH

**Fig. 9:**
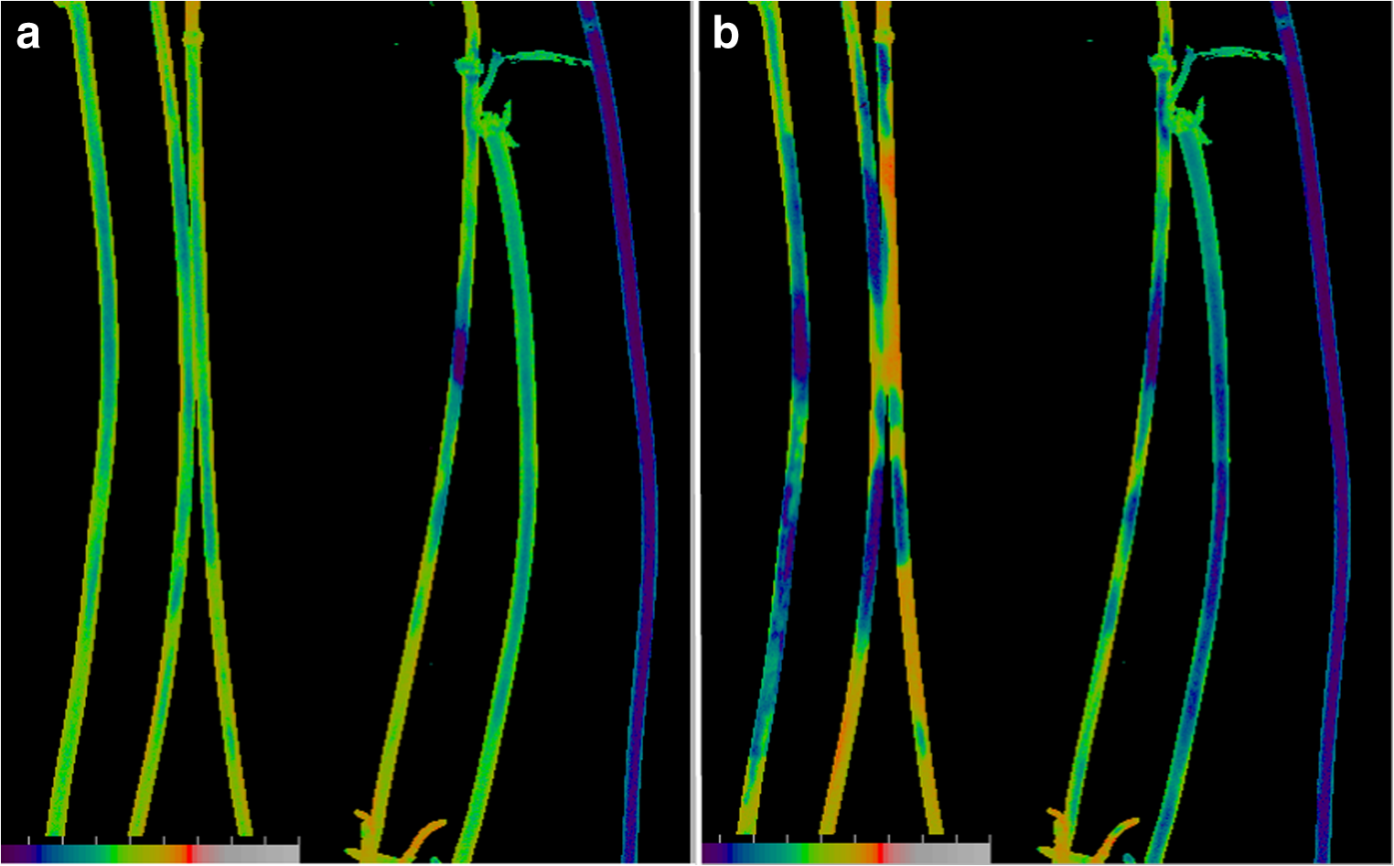
Effective quantum yield of PSII, Y(II) in *Chara* internodes. Images were taken **a** in the beginning of exposure under actinic light (40 μmol m^−2^ s^−1^), before the formation of extracellular pH pattern and **b** after 15 min actinic illumination sufficient for the formation of patterns of external pH and photosynthetic electron transport, Y(II). The colour codes for Y(II) are as follows: violet 0–0.20, dark blue 0.23–0.24, blue to pale blue 0.25–0.38, green 0.40–0.50, yellow green 0.55, orange pale 0.60, orange intense 0.64–0.65, red 0.67–0.69 and pink ≥ 0.7. Note the shared bands of high and low Y(II) in closely contacting internodes. The cells were bathed with AFW containing 0.1 mM KCl, 1 mM NaCl and 0.1 mM CaCl_2_

The formation of shared acid and alkaline zones in closely positioned internodes resulted also in coordination of photosynthetic activity in neighbouring cells. This is seen in Fig. 9a, b, where the images of the effective PSII quantum efficiency Y(II) are presented. The part a shows a homogenous distribution of Y(II) in the beginning of experiment when incubation time was insufficient for the formation of the pH pattern. The cell on the right side of the image, showing very low Y(II), was impaired in photosynthetic activity, even though its bad physiological condition was not evident upon visual inspection.

The prolonged (15 min) incubation of cells under actinic irradiance (40 μmol m^−2^ s^−1^) was accompanied by the formation of non-uniform Y(II) pattern in photosynthetically competent cells but had no influence on the cell with low Y(II) (Fig. 9b). Two features are remarkable. The first one is that the creation of acid bands elevated Y(II) above the level observed before the formation of banding pattern, whereas the drop in Y(II) was observed in cell regions surrounded by alkaline zones. Previous studies documented that photosynthetically active cell regions having elevated Y(II) are attributed to the acid zones, whereas the positions of cell regions with low Y(II) coincide with the alkaline (Krupenina et al. 2008). The second point is that the “photosynthetic bands” are shared in closely contacting internodal cells (see patterns of Y(II) in cells 2 and 3 from the left). Thus, the intercellular interactions mediated by proton transport across the plasma membrane affect and lead to matching the intercellular functions, the photosynthetic activity of chloroplasts in particular.

**Fig. 10:**
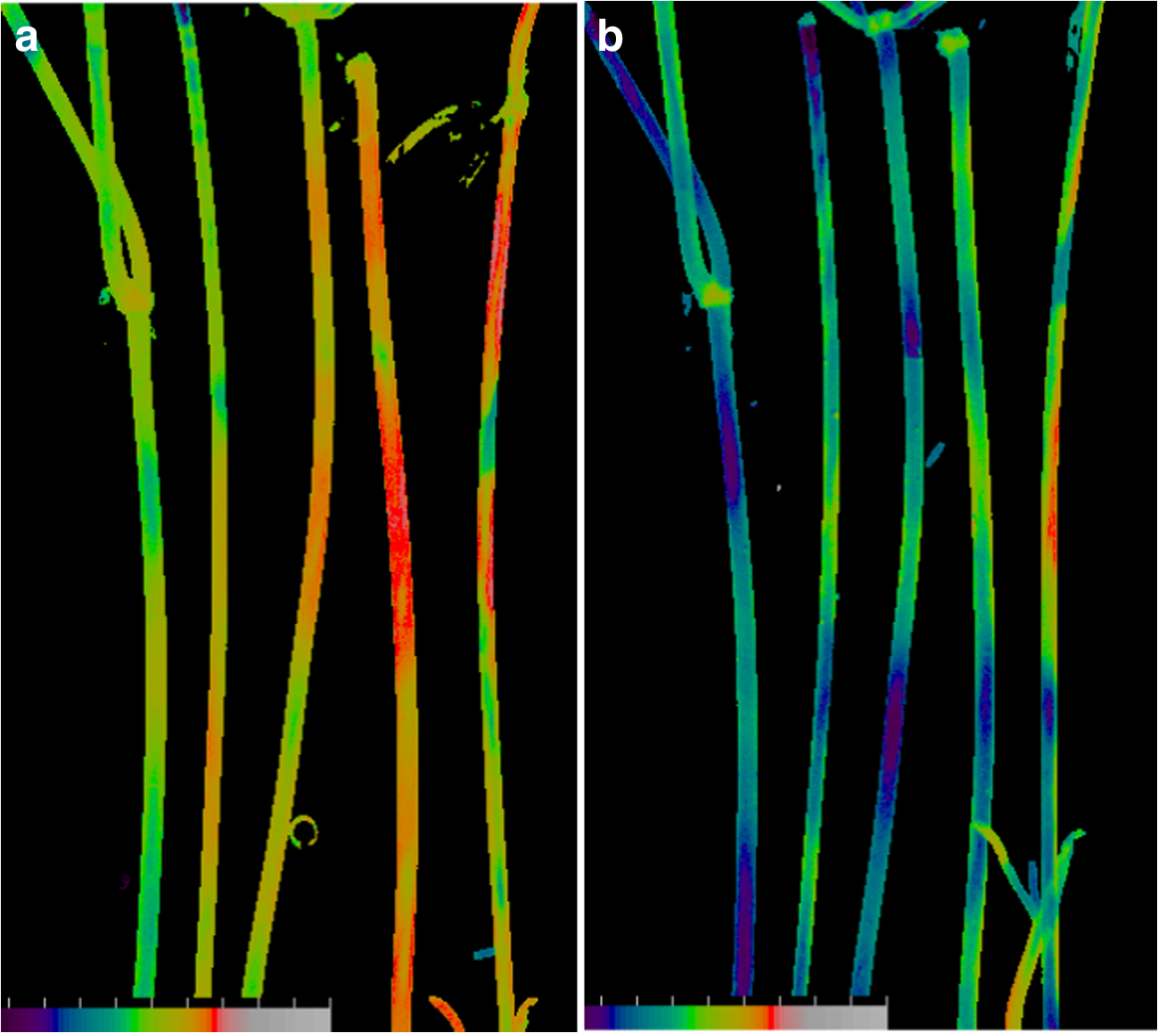
Images of PSII-driven electron flow, Y(II) in *Chara* internodes. Cells were bathed in **a** AFW adjusted to pH 6.1 with 10 mM MES buffer and **b** AFW adjusted to pH 8.8 with 10 mM Tricine-NaOH buffer. For colour codes, see Fig. 9

The influence of external pH on photosynthetic activity was frequently ascribed to different proportions of freely permeable carbon dioxide and impermeable HCO_3_^−^ and CO_3_^2−^ species depending on pH of the medium (Walker and Smith 1977; Lucas 1983; Price et al. 1985; Ray et al. 2003). This view is further supported by Fig. 10. In these experiments, the pH of AFW was adjusted with 10 mM MES buffer to 6.1 and with 10 mM Tricine–NaOH to pH 8.8. It is seen in Fig. 10a that the Y(II) values at pH 6.1 were comparatively high. The replacement of the slightly acidic medium (pH 6.1) with the weakly alkaline solution (pH 8.8) substantially lowered the Y(II) values, while the pattern of Y(II) was still evident (Fig. 10b).

### Reorganization of the cortical cytoplasm after long-term alignment

**Fig. 11:**
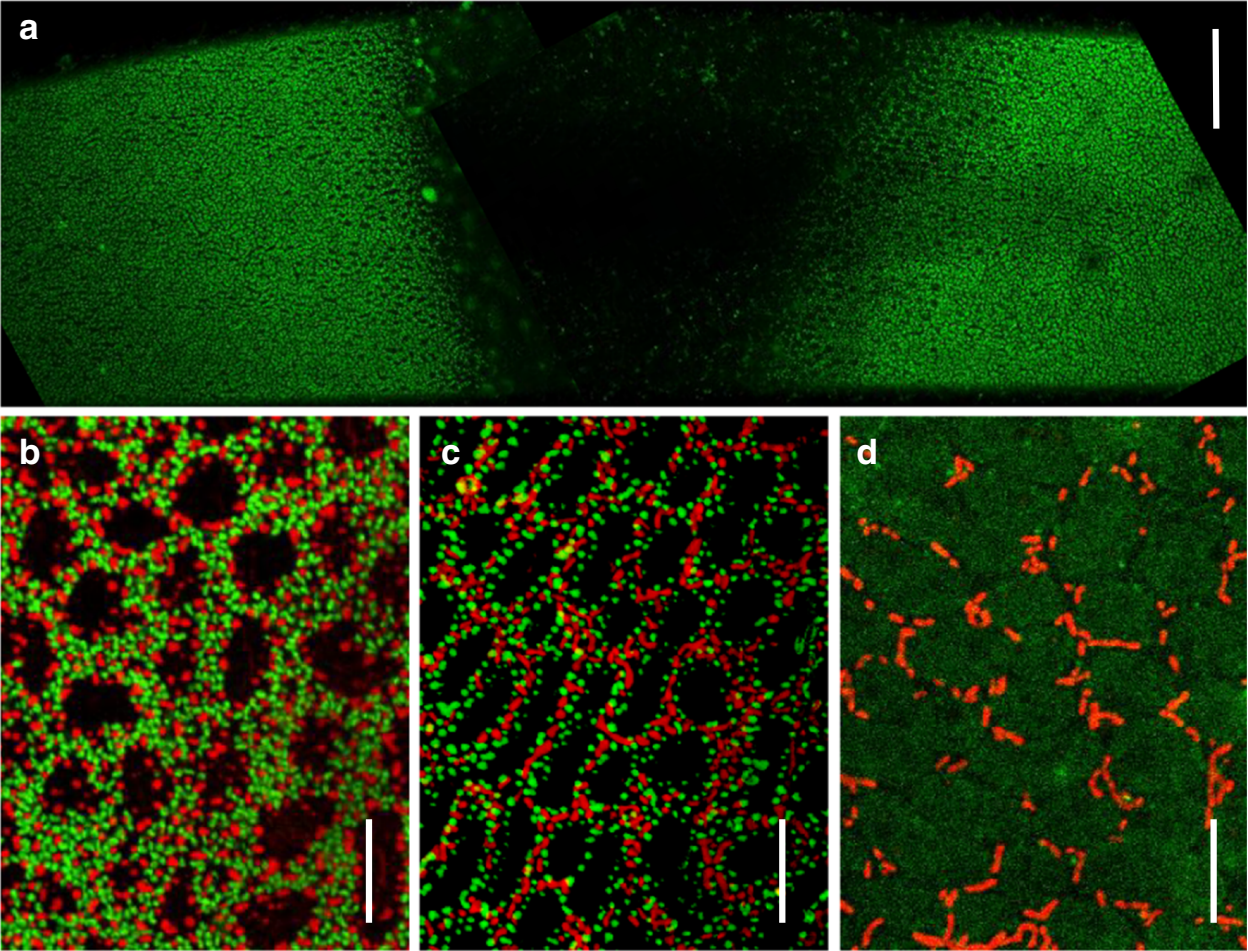
Charasomes (stained with green fluorescent FM1–43) and mitochondria (stained with red fluorescent Mitotracker orange) at and near an induced alkaline band after 3 weeks alignment. **a** The central charasome-free alkaline region is flanked by charasome-rich acidic areas. **b**, **c** Higher magnifications of charasomes and mitochondria in the acid region and **d** in the alkaline band. **a** shows the fluorescence of charasomes only, and **b**–**d** are merged images. Bars are 50 μm (**a**) and 10 μm (**b**–**d**)

In undisturbed, fully grown  internodal cells cultivated under the conditions used in this study, stable patterns of acid and alkaline bands can be correlated with the size and abundance of charasomes and with the size and abundance of cortical mitochondria (see “Introduction” and Fig. 11).

**Fig. 12:**
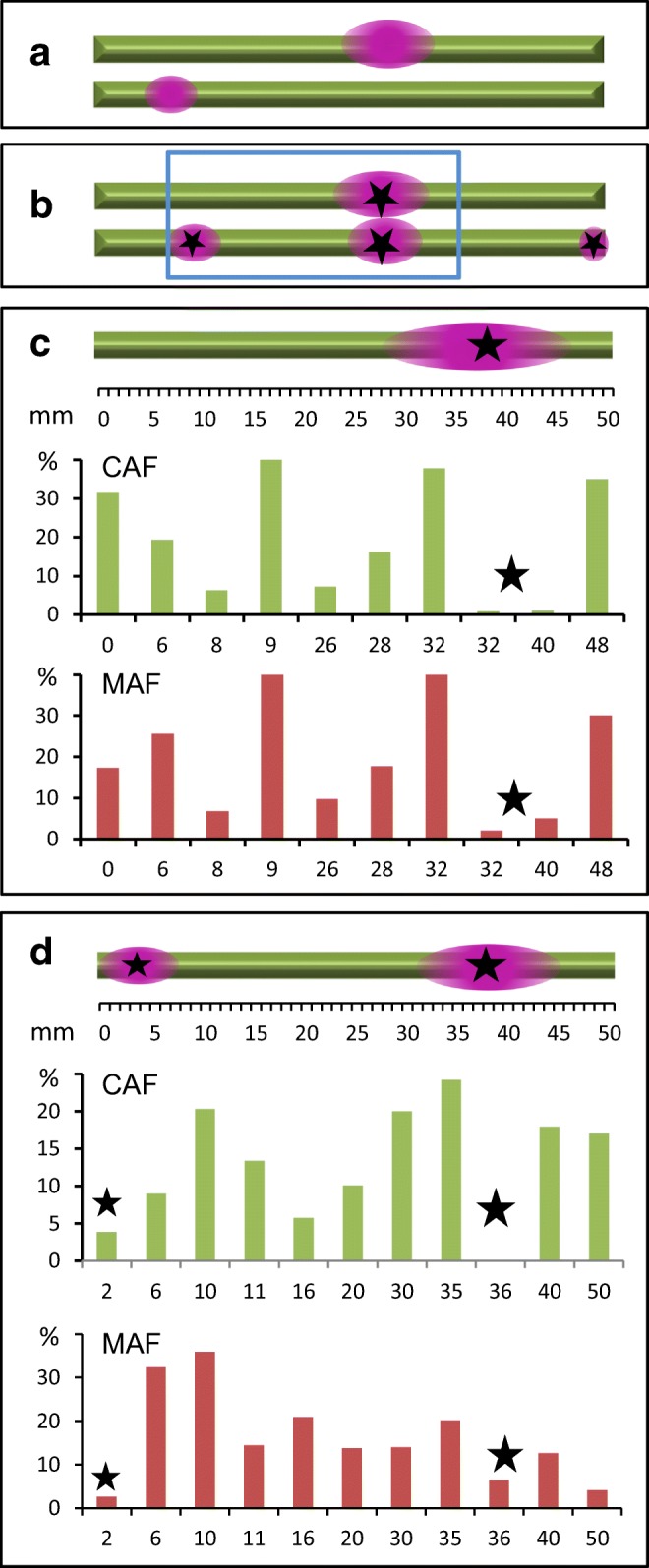
Changes in pH banding pattern and organelle distribution induced by alignment of *Chara* internodal cells. **a**, **b** pH banding pattern before and after alignment for 3 weeks; pink colour indicates alkaline pH. The blue boxed area in **b** was investigated for charasomes and mitochondria, respectively. Large stars mark the positions of confluent alkaline pH bands; small stars mark alkaline regions, which were present on only one cell. **c** Detail of the upper cell, which induced an alkaline band in the neighbour cell. **d** Detail of the lower cell, which acquired a new alkaline band at a previously acidic region. Diagrams show the charasome area fractions (CAFs, green bars) and the mitochondria area fractions (MAFs, red bars) at the positions indicated on the *x*-axis in millimeters. Note that CAFs and MAFs may vary considerably within few micrometres at the acid–alkaline border (e.g. at position 32 in **c**)

In order to find out whether an imposed alkaline pH changes the organization of the cortical cytoplasm, we aligned internodal cells in such a way that an alkaline region was induced in a previously acid band (Fig. S2). Cells were then exposed to a light intensity of 10 μmol quanta m^−2^ s^−1^ during the light cycle (16 h per day). The distribution of charasomes and mitochondria was investigated after 1, 2 and 3 weeks using fluorescent dyes and confocal laser microscopy. Typical examples for such experiments are shown in Fig. 12 and Fig. S3. The size and abundance of charasomes and cortical mitochondria in the upper cell, in which the alkaline band was present before and after alignment, were as described earlier (Schmoelzer et al. 2011; Absolonova et al. 2018b). Charasomes and cortical mitochondria were larger and more abundant at the acid regions than at the alkaline bands at the beginning of the experiment and at its end (compare Fig. 11).

The alkaline band of the upper cell in Fig. 12a almost immediately induced the appearance of an alkaline band in the previously acid region of the lower cell (Fig. 12a, b; see above), but the number and size of charasomes and mitochondria decreased only slowly (data not shown). After 3 weeks, however, the induced alkaline bands became essentially charasome-free with charasome area fractions (CAFs) declining to ≤ 4% (Figs. 11a, d and 12d; Fig. S3). In the acidic regions of both cells, the CAFs varied mostly between 10 and 40%. In spite of the higher variability of the CAFs in the acidic bands, differences in the mean values between acidic and alkaline bands were highly significant (Fig. S4). The size and abundance of cortical mitochondria were similar to that of the charasomes, and along with the local degradation of charasomes, cortical mitochondria became less abundant and the mitochondrial area fraction (MAF; percentage of cell surface occupied by mitochondria) declined (Fig. 12d). In contrast to charasomes, however, mitochondria were never completely absent from the alkaline regions (Figs. 11d and 12d).

The degradation of charasomes cannot be followed in the same cell because of the harmful effects of laser scanning. It is therefore possible that charasome-free regions existed prior to the induction of an alkaline band. In a recent study, we used the pH indicator fluorescein isothiocyanate (FITC) which has a higher spatial and temporal resolution than phenol red and compared it with the distribution of charasomes (Absolonova et al. 2018b). This work revealed the presence of small charasome-free regions which cause alkaline spots or patches that are too small to induce a colour change in phenol red. But the study also confirmed that charasome-free areas with a diameter of ≥ 100 μm were always associated with an alkaline band that could be visualized with phenol red. Small charasome free areas were detected in the “acidic” regions of the cells investigated in the present study (e.g. at positions 8 and 26 in Fig. 12c), and some of them were probably present in the area where an alkaline band was induced. However, the induced alkaline bands, which were inspected after 3 weeks alignment, had a size between 0.3 and 1 cm and the whole alkaline region was more or less charasome-free (Fig. 11a; Fig. S3). If such extended charasome-free areas had been present before alignment, they would have been visible with phenol red.

Quantification of cortical organelles in relation to the pH banding pattern as shown in Fig. 12 and Fig. S3 was performed with eight cells (four pairs). Further 10 cells (5 pairs) were inspected for the presence of charasomes in the joint alkaline bands after 3 weeks alignment. All joint alkaline bands (total number = 22; 10 of them induced) were essentially free of charasomes (CAFs ≤ 5). Therefore, the induction of a new alkaline band at a previously acidic region caused a significant degradation of charasomes in all cells investigated.

## Discussion

### Dynamics of band formation

The dynamics of band formation has hitherto been studied using pH sensitive microelectrodes (e.g. Lucas 1975; Fisahn et al. 1992; Bulychev et al. 2001c, 2003) or water-soluble fluorescent dyes (Absolonova et al. 2018a, b). Both methods measure the pH outside the cell wall in the aqueous surroundings of the cell. In this study, we used 4-heptadecylumbiliferone, a lipophilic fluorescent dye that incorporates into the plasma membrane, hence monitoring the pH at its origin. The analysis of the data obtained with this method shows that the development of a localized patch is distinguished by the propagation of a sharp front marking the rise of the local pH (Fig. 4a). Such a soliton-like dynamics reflects the non-linearity of the ionic transport across the membrane, which includes passive (diffusion) as well as active (ATP-driven transport) mechanisms (Bulychev et al. 2001c; Bulychev and Krupenina 2008; Marten et al. 2010; Dodonova and Bulychev 2011). Growth and decomposition of a pH patch exhibit different spatial-temporal kinetics. The relaxation occurred with relatively small displacement of the pH front. The width of the patch changes by approximately 20% over a period of time when the maximal intensity completely relaxes. This difference in kinetics suggests that the growth reflects an active, autocatalytic process, whereas decomposition is governed by the diffusive relaxation kinetics (compare Lucas 1975).

### PH pattern matching via local increase in external pH

The number of pH bands depends on cytoplasmic streaming and on light intensity (Lucas and Dainty 1977; Bulychev et al. 2001c). At higher streaming rates and at elevated irradiance, the number of alkaline bands is known to increase. Here we show that the number of bands increases upon a rise in the pH of the medium (Fig. 8). It is known that the membrane conductance becomes considerably elevated at high pH due to a large increase in passive fluxes of H^+^ or OH^−^ along their electrochemical gradients (Bisson and Walker 1980). The matching of the pH banding pattern observed in aligned cells (Figs. 6 and 7) can thus be explained by local activation of H^+^/OH^−^ channels via the alkaline regions of aligned cells. Since passive and active fluxes are linked by circular currents, the increase in passive conductance may promote the H^+^ pump activity. Tazawa (2003) speculated that the large increase in plasma membrane conductance during the action potential is accompanied by the transient activation of the H^+^ pump.

Although an earlier study revealed that H^+^/OH^−^ carriers are uniformly distributed over the plasma membrane surface (Lucas and Dainty 1977), recent research showed that acidic regions with a high abundance of charasomes are unlikely to become alkaline (Absolonova et al. 2018a, b). This would explain why not every alkaline band of a given cell induced a corresponding alkaline zone in the acid region of the neighbour cell (see the incomplete matching in Fig. 6d).

### Intercellular pH interactions coordinate photosynthetic activity of chloroplasts in neighbouring cells

When the internodal *Chara* cells are placed parallel close to each other, the surface pH and photosynthetic patterns are readjusted due to alterations of local pH in overlapping unstirred layers. The increase in pH from slightly acidic (pH 6.5) to the alkaline range (pH 8.5–10) shifts the acid–base equilibrium in aerated solutions toward the lowered content of CO_2_ and the increased concentrations of HCO_3_^−^ and CO_3_^2−^. The permeability of the lipid bilayer to a neutral CO_2_ molecule is approximately 10^6^ times higher than that for the anionic species HCO_3_^−^ (Gutknecht et al. 1977). The *Chara* cell regions residing in acid and alkaline zones experience, respectively, sufficiency or deficiency of the supplied carbon. This facilitates the CO_2_-dependent photosynthetic electron flow in chloroplasts underlying CO_2_-enriched acid zones and retards electron flow under the alkaline bands. These functional distinctions are slightly alleviated by virtue of rapid cytoplasmic streaming. At high-pH zones where (CO_3_^2−^) is abundant, sedimentation of CaCO_3_ takes place, thus lowering (Ca^2+^) near cell surface (De Beer and Larkum 2001).

Apart from these general physicochemical events, there are physiological cell responses, such as the elevation of membrane conductance at high pH due to a large increase in passive fluxes of H^+^ or OH^−^ along their electrochemical gradients (Bisson and Walker 1980). The H^+^ influx (OH^−^ efflux) in the alkaline regions may acidify the cytosol and shift the chloroplast stroma to lower pH, which would inhibit the stromal enzymes and CO_2_ assimilation. Furthermore, at lowered stromal pH and equal transthylakoid pH gradients (ΔpH), the lumenal pH becomes more acidic, which promotes the increased dissipation of chlorophyll excitation energy as heat (non-photochemical quenching; Ruban 2016). All these factors—the lowered availability of CO_2_ and the acidified stroma and lumen—reduce the CO_2_-dependent electron transport in chloroplasts underlying the external alkaline zones.

In illuminated internodes, the passive H^+^ influx (OH^−^ efflux) is coupled to the pump-driven H^+^ extrusion at the neighbouring cell regions. These laterally segregated fluxes produce circulating electric currents between the alkaline and acid zones. The increase in passive conductance should stimulate circular currents driven by H^+^-pump operation, which is favourable for the conversion of HCO_3_^−^ to CO_2_ and, accordingly, for the photosynthetic activity. At elevated irradiance when the high rates of CO_2_ delivery into the cell are required for photosynthesis, the number of alkaline bands is known to increase, which implies the enhanced H^+^ pump activity and high rates of HCO_3_^−^ conversion to CO_2_. Based on similar reasoning, the increase in membrane conductance (Bisson and Walker 1980) and the number of alkaline bands at high pH (Fig. 8) might be a compensatory response to the deficiency of CO_2_ under constant irradiance. The appearance of new high pH zones indirectly indicates the stimulation of H^+^ pump that is needed to overcome the shortage of inorganic carbon supply. The HCO_3_^−^ to CO_2_ conversion might proceed within the cell wall near the plasma membrane under non-equilibrium conditions. It should be noted that the increase in external pH did not always elevate the number of zones with high pH and low photosynthetic activity (Fig. 10). It is thus possible that the combination of high pH and other factors (e.g., irradiance level or the duration of incubation at high pH or a high density of charasomes, see above), rather than high pH alone, underlies the increase in frequency of alkaline bands or patches.

### Altered pH banding pattern reorganizes the cortical cytoplasm

Charasomes and mitochondria are large and abundant only in the acidic regions of internodal cells with a stable pH banding pattern. Since the pH banding pattern is dependent on photosynthesis, charasomes degrade when cells are incubated in continuous darkness (Bisson et al. 1991). After a time period of about 10 days only tiny, widely spaced charasomes remain and they are evenly distributed along the cell surface (Hoepflinger et al. 2017). In the light, degradation of charasomes along the whole cell surface has been induced by treating cells with DCMU, an inhibitor of photosynthesis, or with pH buffers applied at concentrations sufficient to suppress the pH banding pattern (Bisson et al. 1991; Schmoelzer et al. 2011). The data presented in this study show that a significant degradation of charasomes occurs also locally under illumination when a previously acidic region becomes alkaline. Along with the degradation of charasomes, the abundance of cortical mitochondria decreases. Their depletion is due to active movement from the cortex towards the streaming endoplasm (Foissner 2004). The metabolic pathways or the conditions responsible for charasome degradation and depletion of mitochondria are currently unknown while it is likely that the decrease in photosynthetic activity is the primary cause for these changes. A possible candidate is the cytosolic pH (pH_cyt_) which is expected to vary according to the external pH banding (Feijo et al. 1999; Bulychev and Komarova 2017). Changes in pH_cyt_ have been shown to affect exo- and endocytosis (e.g. (Sandvig et al. 1987; Cosson et al. 1989) which are required for charasome formation and degradation and for the reorganization of the actin cytoskeleton (Hepler 2016 for review), supporting vesicle and mitochondrial movement. However, the contribution of proton and associated gradients (e.g. Ca^2+^; Plieth et al. 1997) to various cellular processes is still under dispute and further experiments are required to clarify their effect on the cortical organelles in *Chara*. In any case, our study shows that internodal cells are able to respond locally to external signals/conditions by an extensive reorganization of the cortical cytoplasm. We have shown here that charasomes degrade and mitochondria disappear when a previously acidic band becomes alkaline. In previously alkaline regions, the opposite changes take place, i.e. charasomes grow and mitochondria accumulate at the newly formed acid band. These changes are, however, less easy to detect because of the higher variability of charasome and mitochondria area fractions in the acidic regions (Absolonova et al. 2018b and this study).

## Materials and methods

### Algal material, culture conditions and phenol red staining

Thalli of *Chara australis* R.Br. used for microscopic investigation were grown as described in Schmoelzer et al. (2011). Internodal cells were isolated from the main axis with a small pair of scissors and left in artificial freshwater (AFW; 10^−3^ M NaCl, 10^−4^ M KCl, 10^−4^ M CaCl_2_) until use. For visualization of the pH in the external medium along the cell surface, 50 μM phenol red (phenolsulfonphthalein; Sigma-Aldrich, St. Louis, USA) was added to the AFW or to AFW containing 1.5 × 10^−4^ M NaHCO_3_.

### Cell illumination

Whole-cell illumination was provided by a halogen cold light source KL 2500 LCD (Schott) with intensities varying from 0 to 20 mW cm^−2^. This light source enabled the heterogeneous distribution of surface pH to be formed.

### Fluorescence microscopy of pH sensitive dye

The spatial dynamics of the pH bands was measured using the pH sensitive lipophylic dye 4-heptadecylumbiliferone (Fluka; Steinheim, Germany) and an Axio Observer D1 microscope (Carl Zeiss GmbH, Jena, Germany) equipped with a mercury lamp HBO 100. We used excitation at 375 nm and measured emission in the range from 410 to 480 nm. The cells were incubated in AFW containing 0.2 mM 4-heptadecylumbiliferone for 2 h without illumination and washed in AFW several times prior to imaging. The fluorescence intensity of 4-heptadecylumbiliferone increases linearly from pH 9 to pH 11 (Fromherz 1973).

### PH electrode measurements

Alkaline and acid bands were identified with tip-sensitive antimony pH microelectrodes as described in Bulychev et al. (2001a).

### Chlorophyll fluorescence measurements

A series of images was made using a laboratory-built imaging system described by Vanacker et al. (1998). This system produced images with the number of pixels 512 × 512. The camera was fitted with a 135-mm close-focusing objective (Vivatar). This objective allowed semi-macro images of the sample to be made (area imaged ~ 4 cm × 4 cm).

The images of the PSII quantum efficiency Y(II) (ΔF/Fm′) were produced by pixel-by-pixel manipulation of two digital images of chlorophyll fluorescence, one during steady-state photosynthesis (I_ss_) and one during an irradiance sufficiently intense to reduce all the *Q*_A_ in the leaf (*I*_max_). By taking into account the ratio of the steady-state irradiance and the saturating irradiance, an image of ΔF/Fm′ can be calculated (Vanacker et al. 1998). The fluorescent reference discs were used to normalize the fluorescence recorded in the Fo and Fm images.

### Long-term alignment, in vivo staining of organelles and confocal laser scanning microscopy

In order to find out whether an alignment-induced change in the pH banding pattern induces alterations in organelle distribution, we isolated internodal cells of the main axis and stored them in a Petri dish filled with AFW. After exposure to a light intensity of about 10 μE m^−2^ s^−1^ (16/8 h light dark cycle) for 1 week, cells developed a stable pH banding pattern (if not present before) and the distribution of acid and alkaline bands, identified by phenol red, correlated with the distribution of charasomes as described (Schmoelzer et al. 2011). We then placed two cells each into troughs of a Western blot immunotray filled with AFW which was supplemented by phenol red in order to visualize the pH bands. Care was taken to ensure that an alkaline region of one cell was close to the acid region of the other cell. The stability of this spatial arrangement was ensured by a cotton pad placed near the ends of the two cells (Fig. S1).

For in vivo staining of charasomes, internodal cells were pulse labelled for 5 min with 10 μM green fluorescent FM1-43FX (N-(3-triethylammoniumpropyl)-4-(4-(dibutylamino)styryl)pyridinium dibromide) (Thermo Fisher, Waltham, USA) or green fluorescent AM1-44 (Biotium, Hayward, USA). Mitochondria were stained for 30 min with 5 μM MitoTracker Orange (Thermo Fisher, Waltham, USA) dissolved from a 1-mM stock solution in dimethyl sulfoxide (DMSO). All working solutions were prepared with AFW.

The confocal laser scanning microscopes used for imaging fluorescently labelled charasomes and mitochondria were a Leica (Mannheim, Germany) TCS SP5 coupled to a DMI 6000B inverted microscope and a Zeiss LSM 510 coupled to an Axiovert inverted microscope. Images were taken with a 63× water immersion objective (numerical aperture 1.2). Single sections were used for quantification of charasomes, and projections of two to three sections were used for quantification of mitochondria). Charasome area fractions and mitochondria area fractions (% of cell surface area covered by these organelles) were calculated using ImageJ (https://imagej.nih.gov/). Diagrams were produced in Microsoft Excel (https://products.office.com) and Origin (www.originlabs.com).

## Electronic supplementary material

ESM 1 (DOCX 81 kb)

Figure S1. Growing and decaying alkaline patches visualized with fluorescent 4-heptadecylumbiliferone. (PNG 205 kb)

High Resolution Image (TIF 293 kb)

Figure S2. Alignment of two internodal cells in a Western blot tray filled with AFW containing 0.1 mM phenol red The large star indicates a common alkaline band which formed after 2 hours’ alignment. The small asterisk indicates a smaller alkaline band which is visible only in one cell, probably because of the greater distance to the neighbour cell. The position of the cells is secured by a cotton pad seen at the right side of the image. Bar = 1 cm (PNG 84 kb)

High Resolution Image (TIF 139 kb)

Figure S3. Changes in pH banding pattern and charasome distribution induced by alignment of *Chara* internodal cells. PH banding patterns of two cells before **(A)** and after **(B)** three weeks alignment. Stars mark the positions of confluent alkaline pH bands. Charasome area fractions (blue diamonds, left axis) and pH (red lines, right axis) of the upper **(C)** and the lower cell **(D)** in the blue boxed region in C). Stars indicate joint alkaline bands formed after alignment. The small alkaline region at the right side of the lower cell was out of focus for detection of charasomes. (PNG 103 kb)

High Resolution Image (TIF 187 kb)

Figure S4. Comparison of charasome area fractions (CAFs) at acid and alkaline regions after three weeks alignment. Data from 8 cells were collected in 0.5-10 mm intervals along focusable areas of the cell surface. The box plot shows median values (horizontal lines), mean values (crosses), upper and lower quartiles (boxes), maximum and minimum values (whiskers). Differences between the means are highly significant (t-test). (PNG 48 kb)

High Resolution Image (TIF 100 kb)
