# Supplementary material for: PH-dependent cell–cell interactions in the green alga Chara
Source: Protoplasma. 2019 Jul 31;256(6):1737–51. doi: 10.1007/s00709-019-01392-0 (PMC6820879; doi:10.1007/s00709-019-01392-0)
Supplement: Supplementary file 1 — (DOCX 81 kb) [file 709_2019_1392_MOESM1_ESM.docx]

**PH-dependent cell-cell interactions in the green alga *Chara***

Alexej Eremin^1^ ∙ Alexander A. Bulychev^2^ ∙ Christopher Kluge^1^ ∙ Jeremy Harbinson^3^ ∙ Ilse Foissner^4,*^

^1^Institute of Physics, Otto-von-Guericke-University of Magdeburg, 39016 Magdeburg, Germany, ^2^Department of Biophysics, Faculty of Biology, Moscow State University, Moscow 119991, Russia, ^3^Department of Plant Sciences, University of Wageningen, 6708PB Wageningen, The Netherlands, ^4^Department of Biosciences, University of Salzburg, 5020 Salzburg, Austria; *[ilse.foissner@sbg.ac.at](mailto:ilse.foissner@sbg.ac.at)

# **Supplementary Method**

Spatial-temporal dynamics of the pH patch formation can be described by travelling front separating the regions of high and low pH. The shape of the front is reflected in the fluorescence profiles measured in the experiment. The fluorescence profiles *I*(x,t) can be fit using a step-like function (Fig. S1):

$I\left( x \right)=\frac{I_{0}}{(1+\exp(\frac{x-x_{0}}{w_{0}})}$ Eq. S1

where $x_{0}(t)$ is the position of the centre of the step, $I_{0}$ is the height and $w_{0}$ is a characteristic parameter related to the width of the varying part of the front. A similar expression occurs in various mathematical models involving kink-type solitons such motion of domain walls and topological defects.


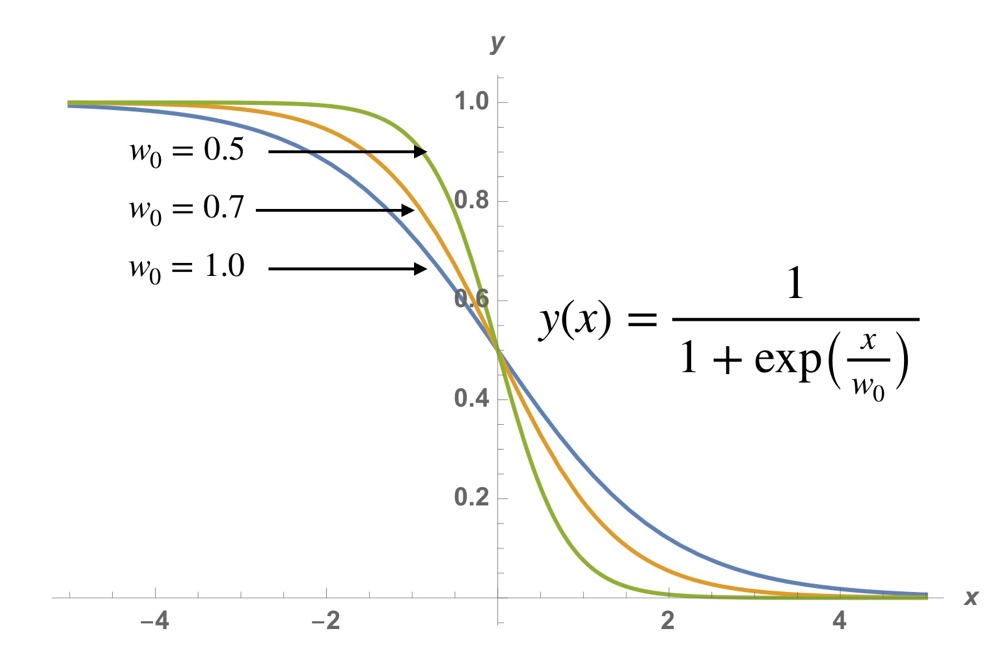


Figure S1. A plot of Eq. S1 for three different values of $w_{0}$.

The dynamics of the pH front exhibits an initial stationary regime where followed by propagation regime, where the front is travelling with a constant velocity. The parameter $w_{0}$remains nearly constant in this regime. The final state is stationary again.
