# Supplementary figures and images for: PH-dependent cell–cell interactions in the green alga Chara
Source: Protoplasma. 2019 Jul 31;256(6):1737–51. doi: 10.1007/s00709-019-01392-0 (PMC6820879; doi:10.1007/s00709-019-01392-0)

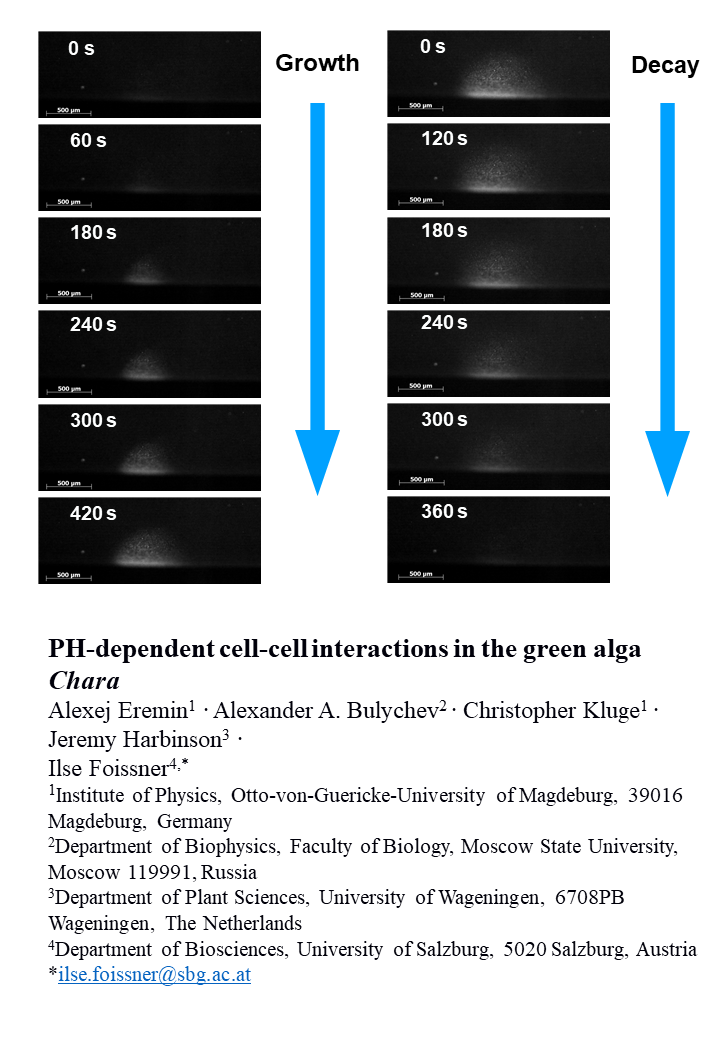

Supplement: Supplementary file 2 — Growing and decaying alkaline patches visualized with fluorescent 4-heptadecylumbiliferone. (PNG 205 kb) [file 709_2019_1392_Fig13_ESM.png]

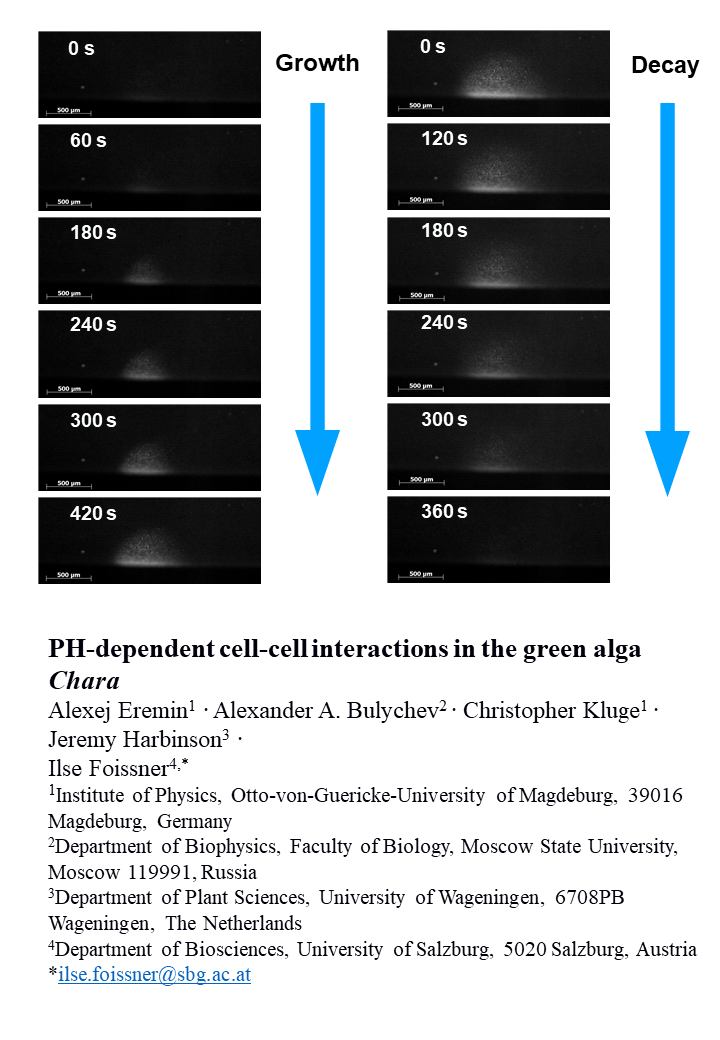

Supplement: Supplementary file 3 — High Resolution Image (TIF 293 kb) [file 709_2019_1392_MOESM2_ESM.tif]

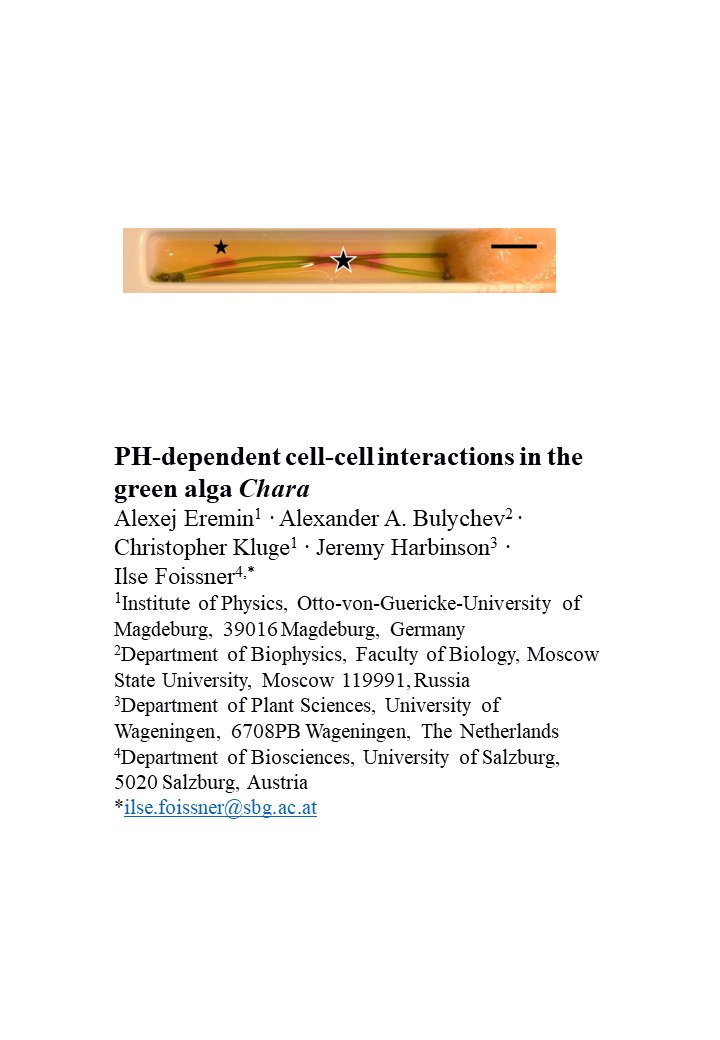

Supplement: Supplementary file 4 — Alignment of two internodal cells in a Western blot tray filled with AFW containing 0.1 mM phenol red The large star indicates a common alkaline band which formed after 2 hours’ alignment. The small asterisk indicates a smaller alkaline band which is visible only in one cell, probably because of the greater distance to the neighbour cell. The position of the cells is secured by a cotton pad seen at the right side of the image. Bar = 1 cm (PNG 84 kb) [file 709_2019_1392_Fig14_ESM.png]

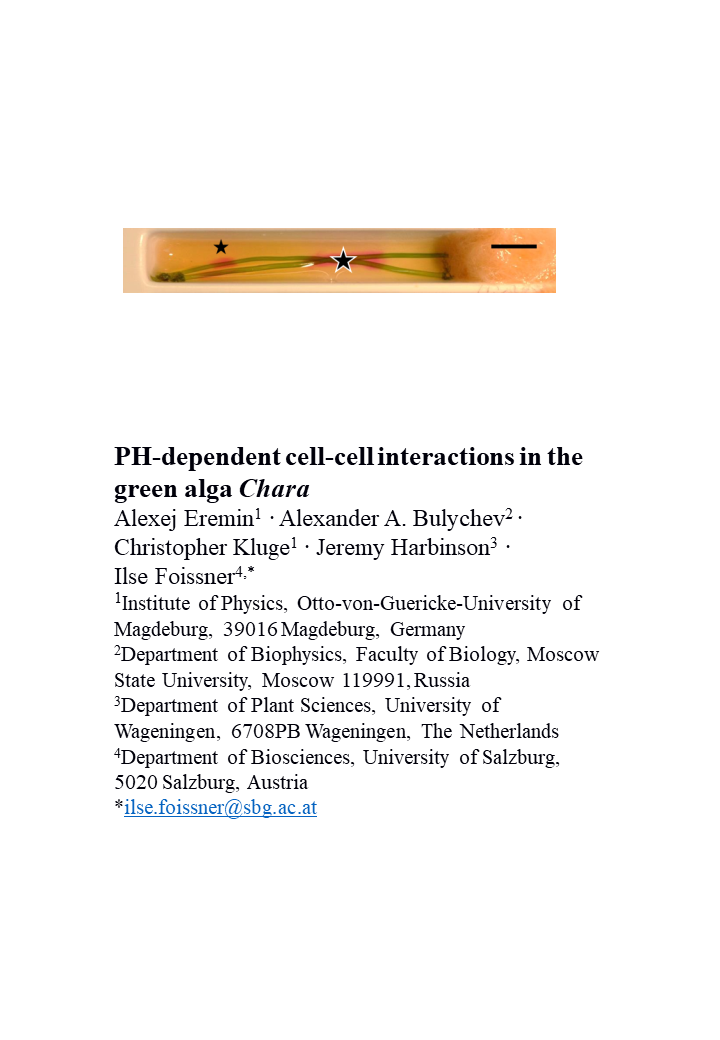

Supplement: Supplementary file 5 — High Resolution Image (TIF 139 kb) [file 709_2019_1392_MOESM3_ESM.tif]

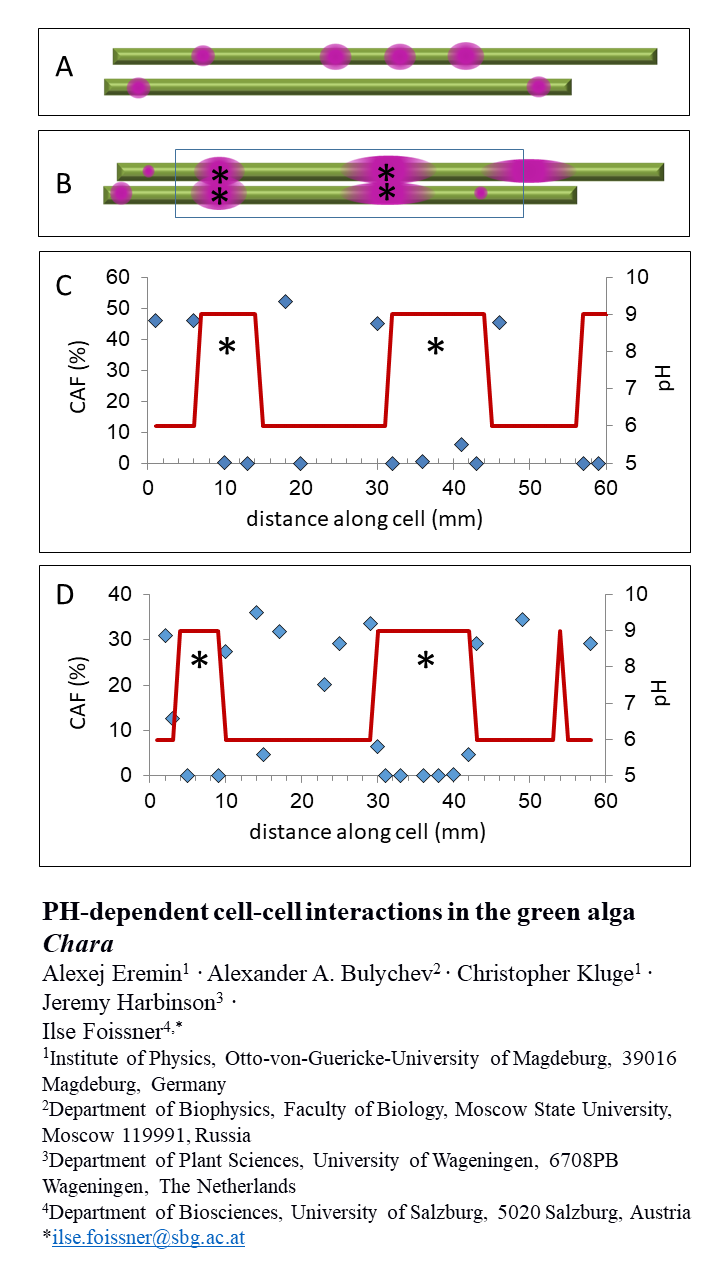

Supplement: Supplementary file 6 — Changes in pH banding pattern and charasome distribution induced by alignment of Chara internodal cells. PH banding patterns of two cells before (A) and after (B) three weeks alignment. Stars mark the positions of confluent alkaline pH bands. Charasome area fractions (blue diamonds, left axis) and pH (red lines, right axis) of the upper (C) and the lower cell (D) in the blue boxed region in C). Stars indicate joint alkaline bands formed after alignment. The small alkaline region at the right side of the lower cell was out of focus for detection of charasomes. (PNG 103 kb) [file 709_2019_1392_Fig15_ESM.png]

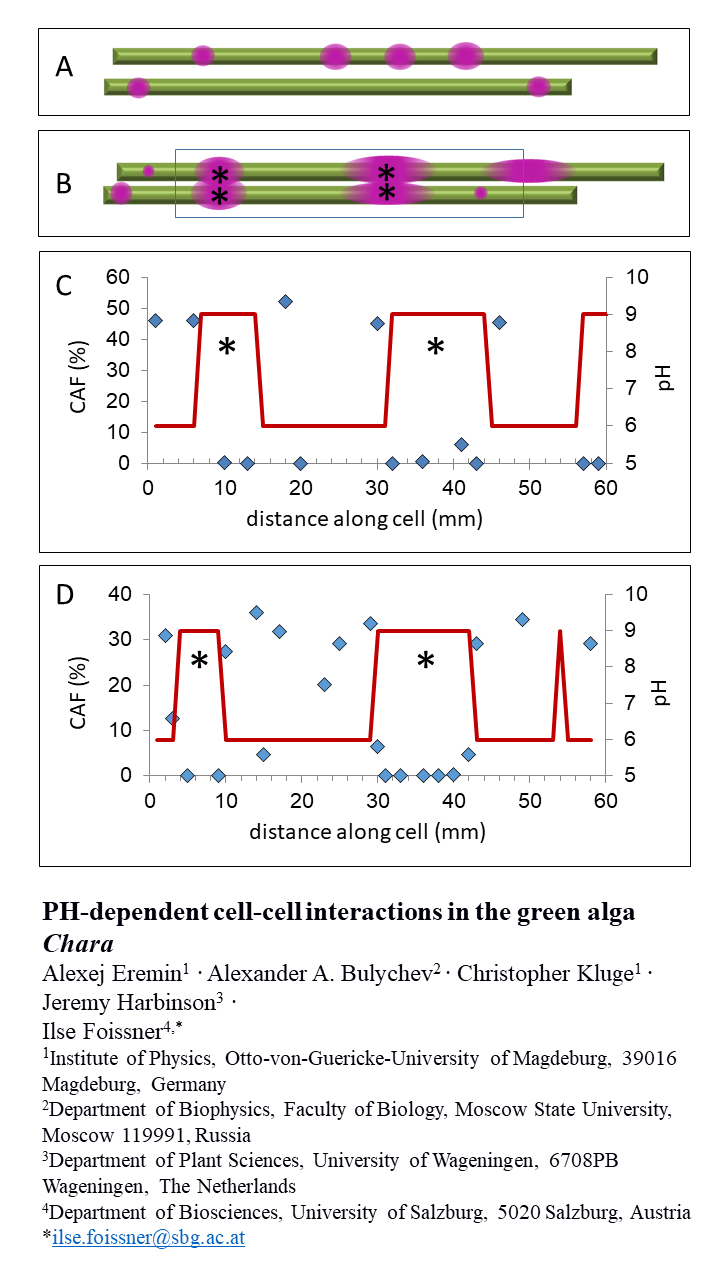

Supplement: Supplementary file 7 — High Resolution Image (TIF 187 kb) [file 709_2019_1392_MOESM4_ESM.tif]

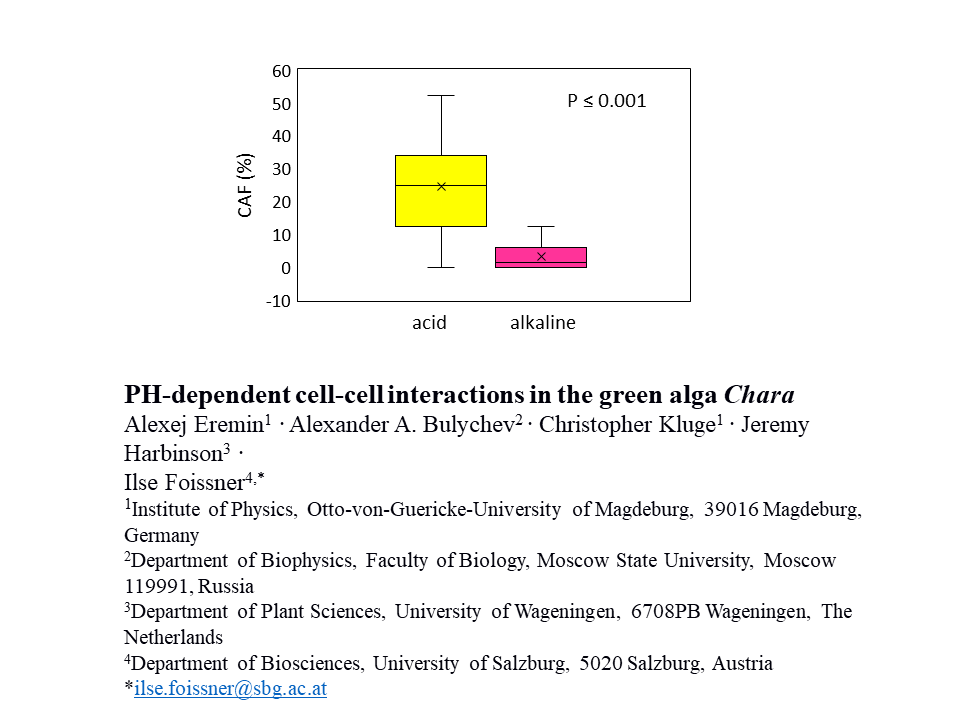

Supplement: Supplementary file 8 — Comparison of charasome area fractions (CAFs) at acid and alkaline regions after three weeks alignment. Data from 8 cells were collected in 0.5-10 mm intervals along focusable areas of the cell surface. The box plot shows median values (horizontal lines), mean values (crosses), upper and lower quartiles (boxes), maximum and minimum values (whiskers). Differences between the means are highly significant (t-test). (PNG 48 kb) [file 709_2019_1392_Fig16_ESM.png]

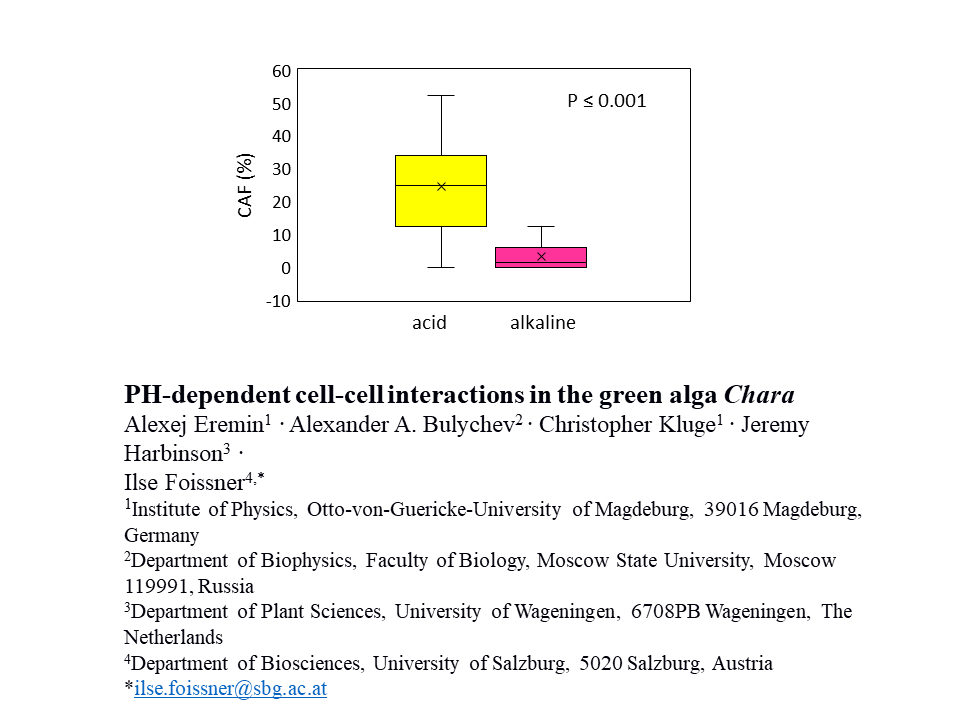

Supplement: Supplementary file 9 — High Resolution Image (TIF 100 kb) [file 709_2019_1392_MOESM5_ESM.tif]
